# Genome-Wide Association Study Provides Insight into the Genetic Control of Plant Height in Rapeseed (*Brassica napus* L.)

**DOI:** 10.3389/fpls.2016.01102

**Published:** 2016-07-27

**Authors:** Chengming Sun, Benqi Wang, Lei Yan, Kaining Hu, Sheng Liu, Yongming Zhou, Chunyun Guan, Zhenqian Zhang, Jiana Li, Jiefu Zhang, Song Chen, Jing Wen, Chaozhi Ma, Jinxing Tu, Jinxiong Shen, Tingdong Fu, Bin Yi

**Affiliations:** ^1^National Key Laboratory of Crop Genetic Improvement, National Sub-Center of Rapeseed Improvement in Wuhan, Huazhong Agricultural UniversityWuhan, China; ^2^Southern Regional Collaborative Innovation Center for Grain and Oil Crops, College of Agronomy, Hunan Agricultural UniversityChangsha, China; ^3^Chongqing Rapeseed Engineering Technology Research Center, College of Agronomy and Biotechnology, Southwest UniversityChongqing, China; ^4^Key Laboratory of Cotton and Rapeseed, Jiangsu Academy of Agricultural ScienceNanjing, China

**Keywords:** *Brassica napus*, association mapping, plant height, haplotype block, selective sweep

## Abstract

Plant height is a key morphological trait of rapeseed. In this study, we measured plant height of a rapeseed population across six environments. This population contains 476 inbred lines representing the major Chinese rapeseed genepool and 44 lines from other countries. The 60K *Brassica* Infinium® SNP array was utilized to genotype the association panel. A genome-wide association study (GWAS) was performed via three methods, including a robust, novel, nonparametric Anderson–Darling (A–D) test. Consequently, 68 loci were identified as significantly associated with plant height (*P* < 5.22 × 10^−5^), and more than 70% of the loci (48) overlapped the confidence intervals of reported QTLs from nine mapping populations. Moreover, 24 GWAS loci were detected with selective sweep signals, which reflected the signatures of historical semi-dwarf breeding. In the linkage disequilibrium (LD) decay range up—and downstream of 65 loci (*r*^2^ > 0.1), we found plausible candidates orthologous to the documented *Arabidopsis* genes involved in height regulation. One significant association found by GWAS colocalized with the established height locus *BnRGA* in rapeseed. Our results provide insights into the genetic basis of plant height in rapeseed and may facilitate marker-based breeding.

## Introduction

Plant height, mainly confined by stem elongation, is a crucial trait that is targeted in modern crop breeding. This notion is best exemplified by the success of the green revolution, which was enabled by the advent of semi-dwarf varieties in cereal crops (Khush, 2001). The short stature and sturdy stalks of semi-dwarf varieties provide plants with enhanced lodging resistance and a greater harvest index, allowing for the more efficient use of fertilizer, pesticide, and water (Khush, 2001; Spielmeyer et al., 2002). Stem elongation is visible when plants transit from the vegetative to the reproductive stage. This process is mediated by multiple phytohormones, including gibberellin (GA), brassinosteroid (BR), and auxin (Wang and Li, 2008). Multiple phytohormone-related genes have been found to affect plant height in the model plants *Arabidopsis* and rice (Wang and Li, 2008). The genetic pathways that determine the height of *Arabidopsis* and rice are fairly conserved (Hong et al., 2005; Wang and Li, 2008; Zhou et al., 2012; Barboza et al., 2013), which makes these pathways informative for the identification of candidate genes in other species.

Rapeseed (*Brassica napus* L., AACC, 2*n* = 38) is the second most important oilseed crop in the world. In view of the major gains in the cereal green revolution, rapeseed breeders are in the progress of modifying plant height to obtain the benefits of improved plant type. Additionally, increasing efforts have been made to identify QTLs for plant height, despite the complexity of polyploids research. One classical approach is linkage analysis that works with experimental, artificially designed populations, created via crossing of selected parents that differ in one or several traits. A large number of QTLs affecting plant height have been identified across the chromosomes in rapeseed (Butruille et al., 1999; Shi et al., 2009; Ding et al., 2012; Qi, 2014; Zhang et al., 2014; Wang et al., 2015a,b; Cai et al., 2016). Liu et al. identified a missense mutation in *BnRGA*, a GA signaling repressor on A06, that causes a semi-dwarf mutant phenotype in rapeseed (Liu et al., 2010).

However, despite a good understanding of the plant height regulation pathway and many of the relevant QTLs, the genetic basis of plant height has not been fully elucidated in rapeseed owing to the limited number of parental lines used in the previous studies. GWAS, an alternative approach that takes advantage of historical recombination events in large populations, provides the opportunity to methodically analyze the genetic architecture of specific traits. Compared with the numerous studies of linkage analysis, association analysis for plant height in rapeseed is rare. Schiessl et al. used 21,623 SNPs from the 60K *Brassica* SNP array to investigate plant height and other traits of 158 European winter-type rapeseed accessions. A total of 69 regions of interest were found to be associated with plant height (Schiessl et al., 2015). Recently, using 19,945 SNPs from the 60K *Brassica* SNP array and a panel of 472 rapeseed accessions, Li et al. detected eight associated regions for plant height, which harbor the candidate genes *GA2ox3, GA2ox8*, and *GA20ox1* (Li et al., 2015).

In addition, another emerging approach is selective sweep analysis, which screens the plant genome for allele frequency differentiation between populations, enabling researchers to identify selected regions conferring favorable phenotypes (Chen et al., 2010). Although few studies have been performed with rapeseed, multiple studies on maize (Hufford et al., 2012), rice (Xie et al., 2015), and cucumber (Qi et al., 2013) have reported successful detection of regions or even genes using selective sweep analysis for target traits. Recently, Xie et al. detected the green revolution *semi-dwarf1* (*sd-1*) as being strongly selected between two rice subpopulations with different proportions of semi-dwarf accessions (Xie et al., 2015).

The objective of this study was to explore the genetic architecture of plant height in rapeseed. We measured the plant height of the association panel in six environments and utilized the 60K *Brassica* Infinium® SNP array to genotype the association panel. Because population size is one determinant affecting the power of association analysis, we enlarged the population size to 520, as compared to previous two association studies as abovementioned, to elevate the detection power. Moreover, in addition to the popular, stringent mixed linear model (MLM), we introduced two permissive, efficient models, the general linear model (GLM), and A-D test, in the present association analysis to improve the detection power. To validate the associated loci, we gathered QTL information from publically available studies and found considerable overlaps with our GWAS loci. We also detected multiple GWAS loci as being strongly selected between two subgroups with different plant height. In the LD decay range up—and downstream of most GWAS loci (*r*^2^ > 0.1), we found plausible candidates orthologous to the documented *Arabidopsis* genes for height regulation.

## Materials and methods

### Plant materials and trait measurement

The association panel used in this study consists of 520 diverse rapeseed accessions derived as part of a recently published study (Xu et al., 2015). The association panel was grown using a randomized complete block design with three replicates on the experimental farms at Changsha (N 28.22°, E 113.00°), Chongqing (N 29.82°, E 106.43°), and Nanjing (N 32.05°, E 118.78°), China, in the 2012/2013, and 2013/2014 growing seasons. The meteorological data of three sites are presented in Table S1. Each line was grown in plots with two rows and 12–15 plants in each row. Five to eight representative plants in the middle of each plot were selected to measure plant height at the BBCH 89 growth stage (fully ripe). Plant height was measured as the height from the base of the stem to the tip of the main inflorescence.

Correlation analysis and variance analysis (ANOVA) of plant height for the association panel across environments were performed with R (Ihaka and Gentleman, 1996). The broad-sense heritability and best linear unbiased prediction (BLUP) of multi-environment phenotypes for this panel were calculated with the R package lme4 (Merk et al., 2012). The broad-sense heritability was calculated as $h^{2}=\sigma_{g}^{2}\text{/}(\sigma_{g}^{2}+\sigma_{ge}^{2}/n+\sigma_{e}^{2}\text{/}nr)$, where $\sigma_{g}^{2}$ is the genetic variance, $\sigma_{ge}^{2}$ is the interaction variance of the genotype with environment, $\sigma_{e}^{2}$ is the error variance, *n* is the number of environments and *r* is the number of replications. The model for the phenotypic trait was *y*_*ijk*_ = μ+*g*_*i*_+*l*_*j*_+(*gl*)_*ij*_+*b*_*k*(*j*)_+*e*_*ijk*_, where μ is the total mean, *g*_*i*_ is the genetic effect of the *i*^*th*^ genotype, *l*_*j*_ is the effect of the *j*^*th*^ environment, (*gl*)_*ij*_ is the interaction effect between the *i*^*th*^ genotype and the *j*^*th*^ environment, *b*_*k*(*j*)_ is the block effect within the *j*^*th*^ environment, and *e*_*ijk*_ is the residual error. All effects were treated as random. The BLUPs and individual environment data were used as phenotypes for the association analysis (Data S1).

### Population structure, kinship, LD decay, and haplotype block analysis

The raw genotypic data generated through the Illumina Infinium platform were further analyzed using the Genome Studio software, which performed cluster refinement with an optimum accession Call Rate > 0.7; SNP Call Freq > 0.75; Minor Freq > 0.05; AA, BB frequency > 0.03; and GenTrain Score > 0.5. After excluding the SNPs without clearly defined clusters or with multiple loci in the genome, 19,167 high-quality SNPs genotyped across 520 rapeseed accessions were utilized to calculate the Q matrix and relative kinship coefficients (K), as previously described (Xu et al., 2015). The parameter *r*^2^ was used to estimate linkage disequilibrium (LD) via TASSEL (Bradbury et al., 2007). The locally paired scatterplot smoothing in R was used for graphical representation of the LD curves (Figures S1–S6). The software Haploview was used to estimate the haplotype block structure in the 520 rapeseed accessions with 19,167 high-quality SNPs (Barrett et al., 2005). The method followed the block definition previously described by Gabrial et al. who defined “strong LD” as a one-sided upper 95% confidence bound on D′ of >0.98 and a lower bound >0.7 (Gabriel et al., 2002). Because the Haploview software computes LD statistics for markers within 500 kb by default, we adjusted this statistic to “0” to force computation for all marker pairs, in case of strong LD between markers beyond 500 kb.

### Genome-wide association study

Trait-SNP association analysis was performed using three methods. The GLM took into account the population structure as a fixed effect. On this basis, the MLM incorporated kinship as the random effects to further eliminate the excess of low *p*-values (Yu et al., 2006). The A-D test was a nonparametric test that included no correction for the population structure. The GLM and MLM were implemented in TASSEL (Bradbury et al., 2007), and the A-D test was performed with the R package ADGWAS (Yang et al., 2014). The uniform Bonferroni threshold for the significance of associations between SNPs and traits was *P* < 5.22 × 10^−5^ (*P* = 1/n, where n = marker number, −log_10_ (1/19,167 = 4.3), which has been widely adopted in the literature (Li et al., 2013; Yang et al., 2014; Liu et al., 2016). The false discovery rate (FDR) analyses were performed using the QVALUE package in R (Storey, 2002). The Manhattan and QQ plots were drawn using the R package qqman. Stepwise regression was performed to estimate the phenotypic variation explained by multiple SNPs using the lm function in R (Ihaka and Gentleman, 1996).

### QTL alignment for plant height between different populations

The QTL information for plant height was collected from documented mapping populations, including seven linkage populations, TN DH (Shi et al., 2009), BE DH (Ding et al., 2012), KN DH (Wang et al., 2015b), QB DH (Qi, 2014), GP DH (Wang et al., 2015a), 8M DH (Zhang et al., 2014), and HJ DH (Cai et al., 2016), and two association panels with 472 and 158 accessions (Li et al., 2015; Schiessl et al., 2015). To align these QTLs to the *B. napus* reference genome (Chalhoub et al., 2014), we performed a BLASTN search against the *B. napus* reference genome with the primer sequences of their flanking markers (Altschul et al., 1990). For traditional markers with left and right primers, such as simple sequence repeat (SSR), the PCR products were generally 100–500 bp in length; thus, only these markers, the left and right primers of which were blasted to the same chromosome and less than 500 bp in distance, were retained for further analysis.

### Regions under selection for plant height

Genome-wide screening for selection was performed using XP-CLR, a method based on modeling the likelihood of multilocus (a set of contiguous markers) allele frequency differentiation between two populations (Chen et al., 2010). We used a 0.08 cM sliding window with 50 bp steps across the whole genome. The maximum number of SNPs assayed in each window was 100, and the command line was XPCLR—xpclr genofile1 genofile2 mapfile outputfile—w1 0.08 100 50 1—p0 0.7. The genetic distances between SNPs were interpolated according to their physical distances in a high-density genetic map of a DH population (unpublished). Final estimates were tabulated in non-overlapping 10 kb windows across the genome, assigning each 10 kb window the mean XP-CLR score calculated by the program. Windows with the top 0.6% of XP-CLR values were selected and merged into regions.

### Candidate gene mining

To define regions of interest that contain potential candidate genes, the local LD decay range was calculated within the flanking regions up to 12,000 kb on either side of the significant SNPs via TASSEL (Bradbury et al., 2007), and a cut-off of 0.1 was used for the LD statistic *r*^2^. Genes within the LD decay range (*r*^2^ > 0.1) were characterized using the software Blast2GO with the default settings (Götz et al., 2008). Genes with gene ontology (GO) terms concerning gibberellin, brassinosteroid, and auxin were highlighted. Then, we used BLASTN searches against the *Arabidopsis* genome to determine whether the candidate SNP tagged genome regions contain genes orthologous to the *Arabidopsis* genes with established roles in plant height regulation.

## Results

### Phenotypic variation among accessions for plant height

**Figure 1:**
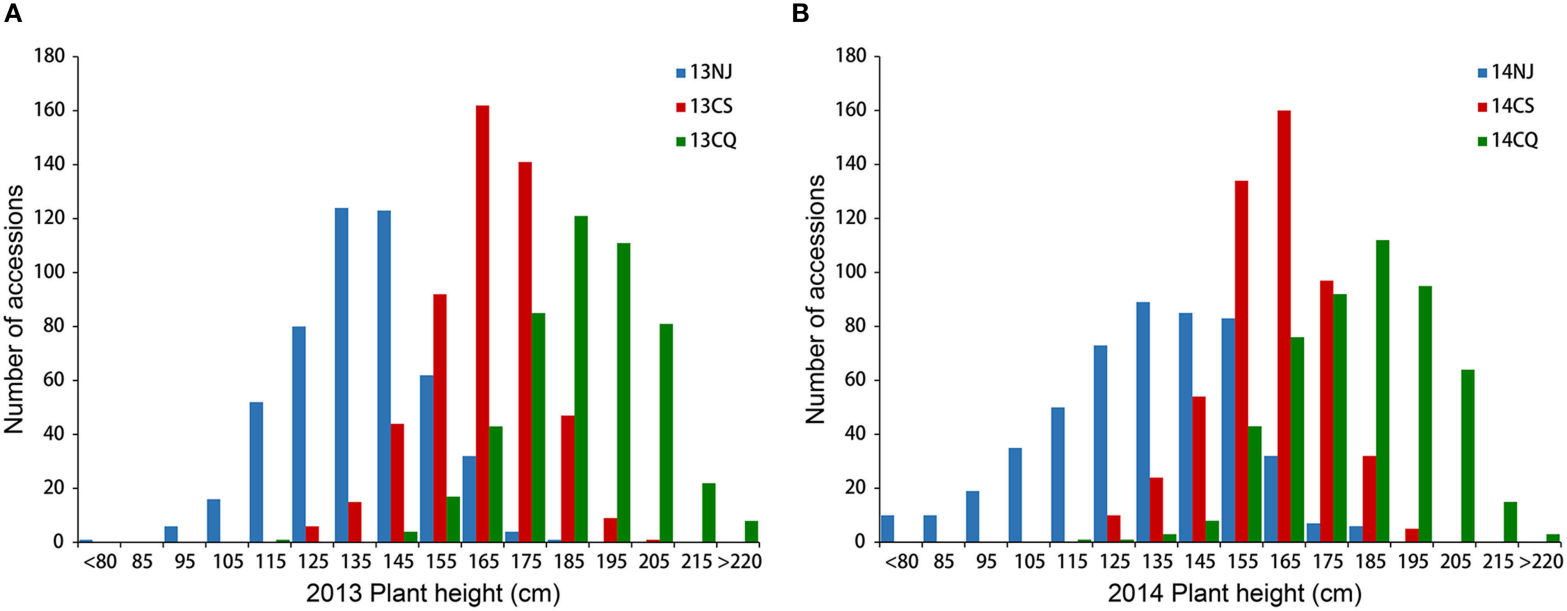
**Distribution of plant height in the association panel of 520 accessions in six environments**. Histogram of three environments in 2013 **(A)** and 2014 **(B)**. 13NJ refers to 2012/2013 Nanjing; 13CS refers to 2012/2013 Changsha; 13CQ refers to 2012/2013 Changsha; 14NJ refers to 2013/2014 Nanjing; 14CS refers to 2013/2014 Changsha; 14CQ refers to 2013/2014 Changsha.

Extensive phenotypic variation was observed for plant height in this association panel. In six environments, plant height varied from 48.33 to 228.39 cm, with an average ranging from 133.55 ± 22.60 to 187.36 ± 15.86 cm (Table 1, Figure 1). The coefficient of variation was relatively constant in the different environments, fluctuating by ~12% (Table 1). Across 2 years, the association panel uniformly exhibited the highest average plant height in Chongqing, the medium height in Changsha and the lowest height in Nanjing (Figure 1). A two-way ANOVA showed that the genotype (G), environment (E) and genotype × environment interaction (G × E) had significant effects on plant height (*P* < 0.001), suggesting the indispensable role of environment in plant height regulation (Table S2). On the other hand, significant (*P* < 0.001) positive correlations between genotypes across six environments were also observed, with the Pearson's correlation coefficients ranging from 0.32 to 0.71 (Table S3). The broad-sense heritability of plant height was 85.19%, based on the phenotypic data measured in six environments. The results indicated that despite the environmental effects, plant height for a given genotype is fairly stable.

**Table 1:** **Phenotypic variation of plant height in the association panel**.

**Environment**	**Min (cm)**	**Max (cm)**	**Mean ± SD (cm)^a^**	**CV (%)^b^**
2012/2013 Nanjing	74.3	183.3	137.1 ± 16.1	11.8
2013/2014 Nanjing	48.3	187.7	133.6 ± 22.6	16.9
2012/2013 Changsha	115.2	202.6	165.3 ± 17.1	10.4
2013/2014 Changsha	120.6	194.4	161.4 ± 18	11.2
2012/2013 Chongqing	117.3	228.4	187.4 ± 15.9	12.7
2013/2014 Chongqing	117.8	224.9	181.8 ± 17.6	12.9

a *SD standard deviation*.

b *CV coefficient of variation*.

### Haplotype blocks

**Figure 2:**
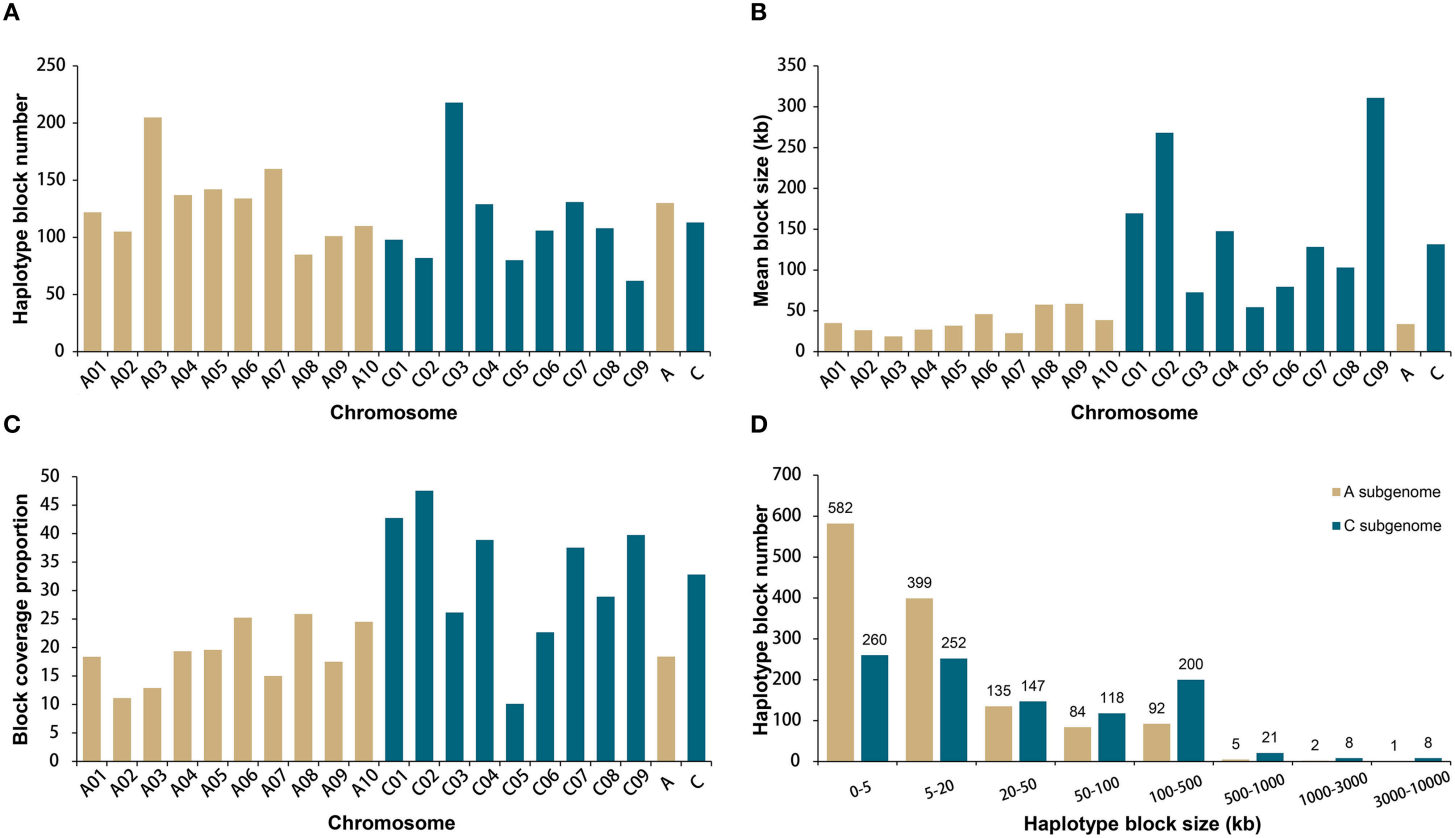
**Comparative analysis of haplotype blocks in the A and C subgenomes of 520 rapeseed accessions. (A)** Haplotype block number on the A and C subgenome chromosomes. **(B)** Mean haplotype block size on the A and C subgenome chromosomes. **(C)** Haplotype block coverage proportion on the A and C subgenome chromosomes. **(D)** Size range distributions of the haplotype blocks in the A and C subgenomes.

A total of 19,167 high-quality SNPs throughout the genome were used to estimate haplotype blocks in our association panel (Data S1). A total of 2315 conserved haplotype blocks were detected across all rapeseed accessions, spanning 177.4 Mb (20.9% of the assembled *B. napus* genome, Table S4). Despite the subtle difference in mean number, 130 for A and 113 for C subgenome chromosomes, the haplotype blocks in A subgenome accounted for less than a quarter (24.8%) of the total block size, with an average of 33.8 kb, whereas the blocks in C subgenome attributed more than three-fourths (75.2%), with an average of 131.7 kb (Figures 2A,B). Correspondingly, higher block coverage proportions were observed on major C subgenome chromosomes (32.8% on average, Figure 2C) than on A subgenome chromosomes (18.4% on average, Figure 2C).

We identified 9 blocks larger than 3 Mb in size, which accumulatively accounted for more than one-quarter (27.4%) of the total block size and nearly one-tenth (5.7%) of the assembled *B. napus* genome (Table 2, Figure 2D). All but one block was located on C subgenome chromosomes (Table 2). A recent study identified the physical locations of 19 *B. napus* chromosome centromeres (Mason et al., 2015). We found that 6 of 9 large blocks (>3 Mb) span across or locate near their corresponding centromeres, including the blocks on A06, C01, C02, C04, C07, and C08 (Table 2). To further explore the effects these pericentromeric haplotype blocks exerted on chromosomal LD decay, we recalculated the LD measurement parameter r^2^ for A06, C01, C02, C04, C07, and C08 after excluding the SNPs located in these blocks (Figures S1–S6). In this analysis, the LD decay of the C subgenome chromosomes decreased dramatically to less than 3 Mb when *r*^2^ = 0.1, and the majority fluctuated at ~2000 kb (Figures S2–S6).

**Table 2:** **Location and size of the large haplotype blocks of the association panel and their corresponding centromeres**.

**Chromosome**	**Centromere**		**Centromere size (Mb)**	**Haplotype block**		**Block size (Mb)**
	**Start (Mb)**	**End (Mb)**		**Start (Mb)**	**End (Mb)**	
A06	11.1	11.1	0.02	11.1	14.5	3.3
C01	17.9	24.2	6.2	18.0	26.2	8.2
C02	31.8	32.2	0.3	23.3	31.9	8.6
C04	17.1	19.4	2.3	15.4	21.6	6.3
C07	5.4	7.2	1.8	5.2	8.2	3.0
C07	5.4	7.2	1.8	13.4	18.1	4.7
C08	5.8	6.4	0.6	6.4	9.5	3.1
C09	23.1	23.4	0.3	24.7	31.7	7.0
C09	23.1	23.4	0.3	33.4	37.7	4.3

### Genome-wide association study

**Figure 3:**
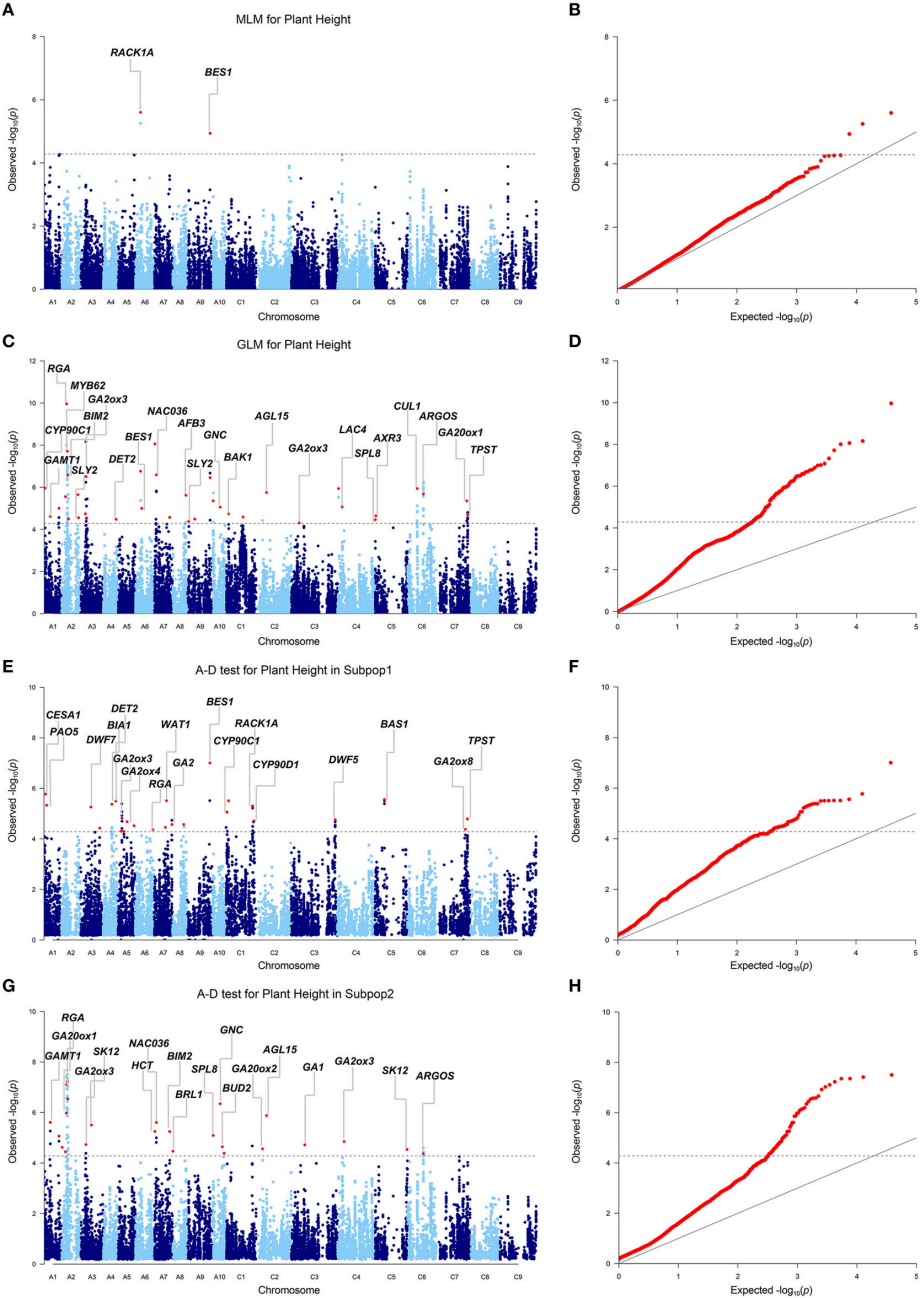
**Genome-wide association study of plant height in the panel of 520 accessions. (A)** Manhattan plot of the MLM for plant height. **(B)** Quantile-quantile plot of the MLM for plant height. **(C)** Manhattan plot of the GLM for plant height. **(D)** Quantile-quantile plot of the GLM for plant height. **(E)** Manhattan plot of the A-D test for plant height in subpopulation 1. **(F)** Quantile-quantile plot of the A-D test for plant height in subpopulation 1. **(G)** Manhattan plot of the A-D test for plant height in subpopulation 2. **(H)** Quantile-quantile plot of the A-D test for plant height in subpopulation 2. The dashed horizontal line depicts the Bonferroni significance threshold [–log_10_(*p)* = 4.3]. Peak SNPs are indicated with red dots, and the corresponding candidate genes are annotated. Because multiple candidate genes may be identified for one loci, the most possible candidate gene is annotated in the plots. Full gene information is listed in Table S5.

When we firstly performed GWAS with the stringent MLM using the BLUP values across 6 environments, 3 SNPs significantly associated with plant height were identified at the Bonferroni threshold of *P* < 5.2 × 10^−5^ [1/19,167, −log10(*P*) = 4.3], corresponding to 2 loci located on chromosomes A06 and A09 (Table 3, Figures 3A,B). When we used the less stringent False Discovery Rate (FDR) correction, coincidentally, no additional significant SNPs were detected at the threshold of *Q* < 0.1. When using a simple additive model, the two loci explained 9.2% of the phenotypic variation.

**Table 3:** **Summary of the genome-wide significant associations for plant height identified by the MLM**.

**SNP^a^**	**Chr**.	**Position (kb)**	**Allele**	**MAF^b^**	**MLM^c^**	**GLM^d^**	**A-D^e^**	**QTL^f^**	**XP-CLR^g^**	**Candidate gene**	**Annotation**
Bn-A06-p7023598	A06	6472	A/C	0.38	5.6	6.8			6.7	BnaA06g12310	*RACK1A*
										BnaA06g12520	*BEE1*
Bn-A09-p32172515	A09	30,032	A/G	0.39	4.9	6.5	7 [1]	TN, 158	3.6	BnaA09g44210	*BES1*

a *SNP Only the peak SNP in a defined locus is shown*.

b *MAF Minor allele frequency*.

c *MLM –log_10_(p) value of the SNP detected by MLM*.

d *GLM –log_10_(p) value of the SNP detected by GLM*.

e *A–D –log_10_(p) value of the SNP detected by the A-D test; the number in square brackets indicates the subpopulation in which the SNP was detected*.

f *QTL name of the mapping population*.

g *XP-CLR XP-CLR score of the nearest selection region of the peak SNP*.

Given the large number of accessions and SNPs used in this study, this level of detection power with the MLM was not effective enough. To make better use of the information from the genotyping and phenotyping data in this study, two permissive models, the GLM and A-D test, were applied to the association analysis. Briefly, the two models detected 37 (GLM), and 49 (A-D test) loci significantly associated with plant height BLUP values under the same Bonferroni threshold as above (Table S5, Figures 3C–H). The loci from the GLM and A-D test explained 38.3 and 37.0% of the total phenotypic variance, respectively, when a simple additive model was applied.

We then compared the consistency of the identified loci between the three methods. The two loci detected by the MLM were repeatedly detected by either the GLM or the A-D test. The locus on A09 was significant in the three methods (Table 3). A total of 28 associated loci were consistently detected between the GLM and A-D test, whereas 19 and 31 peak SNPs were exclusive to the two methods, respectively, (Table S5). Furthermore, 100% (2/2), 91.9 (34/37), and 85.7% (42/49) of the loci detected by the MLM, GLM, and A-D test were validated in at least one individual environment in addition to BLUP, suggesting the reliability and repeatability of our results (Table S6). Altogether, the three methods identified 223 SNPs, corresponding to 68 loci, as significantly associated with plant height (Table S5). These loci were unevenly distributed over the chromosomes, except for C08 and C09, and explained 42.3% of the total phenotypic variance. A02 had the maximum 9 loci, followed by 6 in A03, A07, and 5 in A01, A05, and C01. The remaining chromosomes had 2–4 loci (Table S5). Taken together, more than 2/3 of the loci (46/68) were distributed in the A subgenome, and the rest were in the C subgenome.

### QTL alignment for plant height between different populations

Because the two permissive methods, the GLM and A-D test, potentially introduce more false positives than the MLM, we compared our results with QTLs from publically available literature for plant height. Totals of 87, 9, 70, 15, 11, 27, and 21 QTLs were reported in seven linkage mapping populations, including the TN, BE, KN, QB, GP, 8M, and HJ doubled haploid (DH) populations (Shi et al., 2009; Ding et al., 2012; Qi, 2014; Zhang et al., 2014; Wang et al., 2015a,b; Cai et al., 2016), and 8 and 69 QTLs were reported in two association panels with 472 and 158 accessions (Li et al., 2015; Schiessl et al., 2015). A total of 289 could be aligned to the *B. napus* reference genome (Table S7).

Among the 68 loci identified through GWAS, 48 loci (70.5%) overlapped the confidence intervals of previously reported QTLs, and 20 loci (29.4%) overlapped the common QTLs from at least 2 mapping populations (Table S8). One example included the locus Bn-A09-p32172515 detected by the MLM on A09, which overlapped the confidence interval of one common QTL from the TN DH and the association panel with 158 accessions (Table 3). Four out of eight loci and 12 out of 69 loci identified in the association panels with 472 and 158 accessions overlapped the confidence intervals of loci in the present study, and three loci Bn-A03-p7672403, Bn-A05-p23020834, and Bn-A07-p21965076 were consistently detected among three association studies (Table S8). For the GLM, and the A-D test, 29 out of 37 (78.3%) and 35 out of 49 (71.4%) loci were validated in previous mapping populations, and three common loci and one loci solely significant in the A-D test were jointly validated by at least three mapping populations (Table 4). The results thus provided additional support for the reliability of our results.

**Table 4:** **Summary of the GWAS loci jointly detected in multiple previous mapping populations**.

**SNP^a^**	**Chr**.	**Position (kb)**	**Allele**	**MAF^b^**	**MLM^c^**	**GLM^d^**	**A-D^e^**	**QTL^f^**	**XP-CLR^g^**	**Candidate gene**	**Annotation**
Bn-A01-p9254925	A01	7912	T/G	0.40		4.6	5.6 [2]	TN, QB, GP	3.2	BnaA01g15540	*GAMT1*
										BnaA01g15350	*GNL*
Bn-A02-p9240182	A02	6043	T/G	0.08			6.0 [2]	KN, 8M, HJ		BnaA02g11210	*GA20ox1*
Bn-A02-p9610453	A02	6418	A/C	0.23		10.0	7.1 [2]	KN, 8M, HJ		BnaA02g12260	*RGA*
Bn-A03-p7672403	A03	6973	T/G	0.46		6.5	4.7 [2]	TN, KN, 472, 158		BnaA03g15880	*GA2ox3*

a *SNP Only the peak SNP in a defined locus is shown*.

b *MAF Minor allele frequency*.

c *MLM –log_10_(p) value of the SNP detected by MLM*.

d *GLM –log_10_(p) value of the SNP detected by GLM*.

e *A–D –log_10_(p) value of the SNP detected by the A-D test; the number in square brackets indicates the subpopulation in which the SNP was detected*.

f *QTL name of the mapping population*.

g *XP-CLR XP-CLR score of the nearest selection region of the peak SNP*.

### Regions under selection for plant height

**Figure 4:**
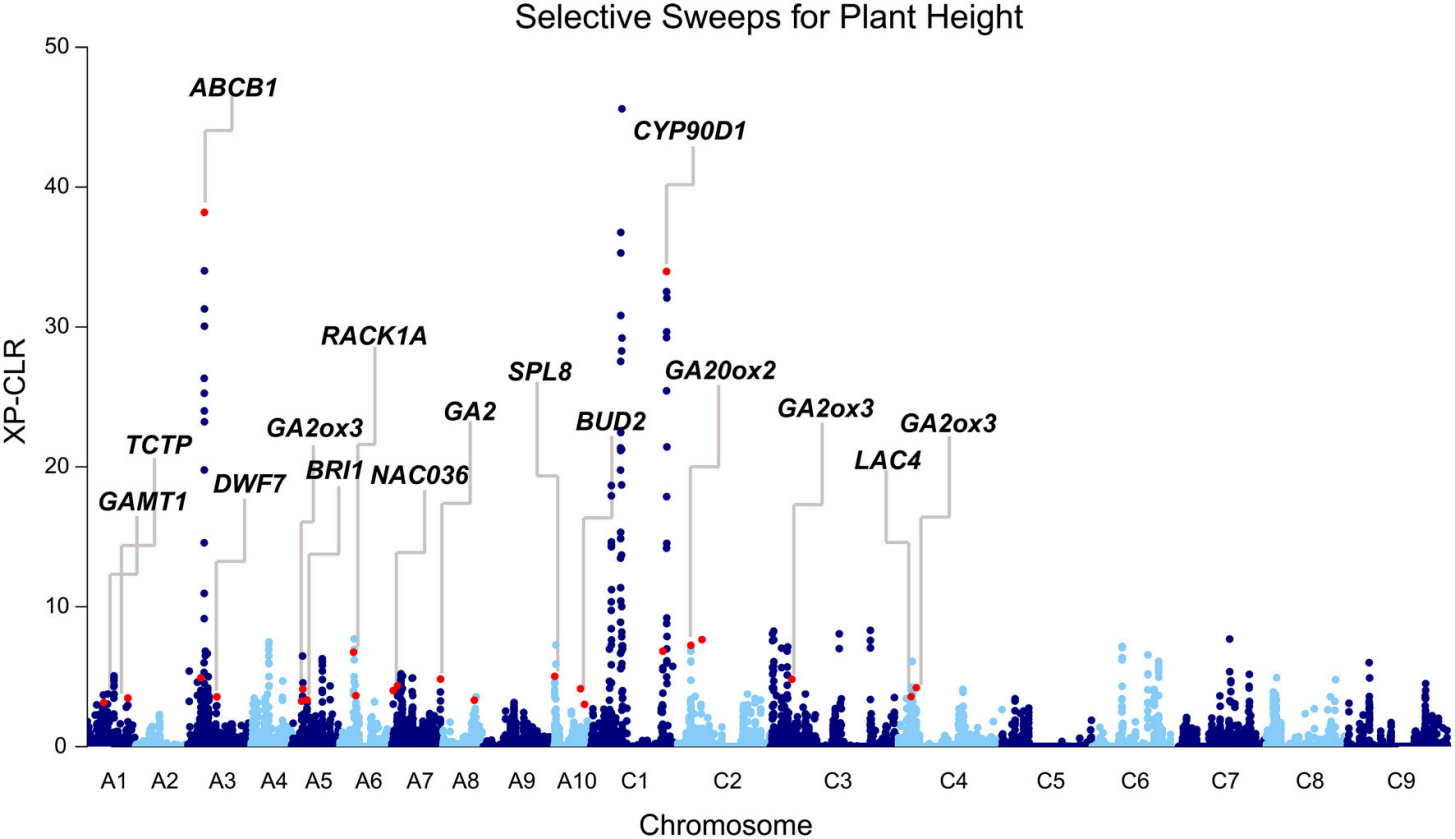
**Differential selection revealed by the genome-wide screening of the selection between Subpop2-dwarf and Subpop2-tall using XP-CLR**. Subpop2-tall is the reference population, and Subpop2-dwarf is the object population. Peak signals overlapping with GWAS loci are indicated with red dots, and the corresponding candidate genes are annotated.

Because favorable alleles can be targeted in the breeding process, we performed the genome-wide selective sweep analysis to identify the signatures of selection for plant height. Using a population structure (Q) matrix value threshold of 0.6, 65, 398, and 57 lines were assigned to subpopulation 1, subpopulation 2, and a mixed subpopulation, respectively. To reduce the potential confounding effects of population structure, we performed the selective sweep analysis in subpopulation 2. The 398 accessions of subpopulation 2 were divided into two groups according to the plant height: Subpop2-dwarf (151, BLUP < 0) and Subpop2-tall (247, BLUP > 0). XP-CLR analysis across the genome was performed in Subpop2-dwarf using Subpop2-tall as the reference population. In total, 107 merged regions (highest 0.6% of the non-overlapping 10 kb segments) were regarded as candidate targets of selection with XP-CLR scores ranging from 3.0 to 45.6 (Table S9, Figure 4). These regions varied from 10 to 250 kb in length (46.5 kb on average) and occupied 5.1 Mb (0.8%) of the assembled *B. napus* genome. Among the 68 loci identified through GWAS, more than one-third of the loci (24) overlapped with the selection regions, including the two loci detected by MLM (Table 3, Figure 4). Furthermore, the peak SNPs of 24 loci were 600 kb away from of the peak signals of selection regions, with an average of 186.6 kb (Table S5). Particularly strong selection was observed in the vicinity of the peak SNPs Bn-A03-p8663803 on A03 and Bn-scaff_15936_1-p270915 on C01 (XP-CLR score = 38.2 and 32.1, respectively, Table S5, Figure 4). These results indicated that these loci were potentially selected in rapeseed semi-dwarf breeding.

### Candidate gene mining

Based on the GO annotation and the publically available literature, we mined for candidate genes within the LD decay range (*r*^2^ > 0.1) up—and downstream of the GWAS loci. Encouragingly, a total of 95 candidate genes for height regulation were predicted for 65 loci. Due to the low rate of LD decay (1084.6 kb on average, *r*^2^ = 0.1), more than one-third (24/65) of the GWAS loci had at least two candidate genes (Table S5).

**Figure 5:**
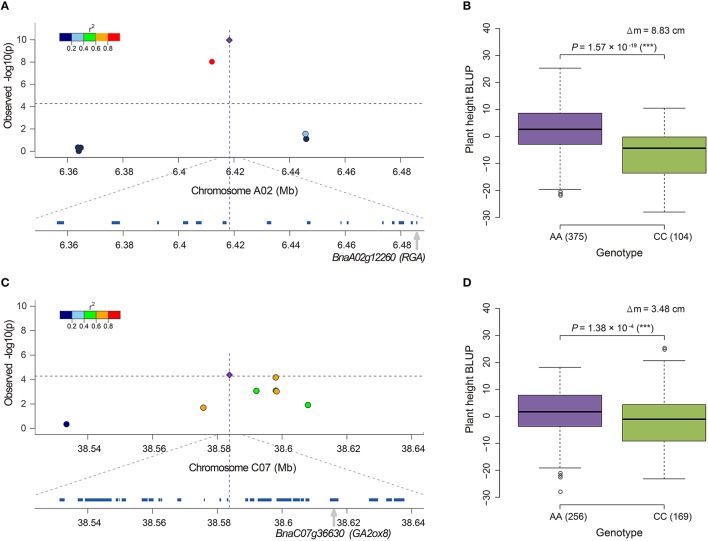
**Candidate genes near the SNPs associated with plant height in rapeseed and phenotypic differences between lines carrying different alleles of these SNPs. (A)** Candidate gene *BnaA02g12260 (RGA)* for locus Bn-A02-p9610453 detected by the GLM. **(B)** Phenotypic difference between lines carrying different alleles, AA and CC, of locus Bn-A02-p9610453. **(C)** Candidate gene *BnaC07g36630 (GA2ox8)* for locus Bn-scaff_16069_1-p2212587 detected by the A-D test. **(D)** Phenotypic difference between lines carrying different alleles, AA and CC, of locus Bn-scaff_16069_1-p2212587. In **(A,C)**, the most significantly associated SNP is indicated by a purple diamond. The color coding of the remaining markers reflects the *r*^2^ values with the peak SNP. The dashed horizontal line depicts the Bonferroni significance threshold [−log_10_(*p)* = 4.3]. In **(B,D)**, the difference of mean (Δm) and the *P*-value based on ANOVA are also given. ^*^significant at *p* ≤ 0.05; ^**^significant at *p* ≤ 0.01; ^***^significant at *p* ≤ 0.001. The numbers in brackets behind AA or CC refer to the number of accessions carrying the corresponding allele.

Notably, 36.8% (35/95) of the candidate genes were annotated to associate with GA, and 20.0% (19/95), and 16.8% (16/95) of the genes were involved in the GA biosynthesis and signaling pathway, respectively (Table S5). *BnaA06g34810* (*BnRGA*) encodes a GA signaling repressor, a missense mutation in the VHYNP motif that causes a semi-dwarf mutant phenotype in *B. napus* (Liu et al., 2010). In the present study, it was located at 169.3 kb downstream from the peak SNP Bn-A06-p23852270 (*r*^2^ > 0.2, Table S5). Another paralog of *BnRGA* on A02, *BnaA02g12260*, displayed the top signal in both the GLM and A-D test (Figures 3C,E). *BnaA02g12260* was located at 67.3 kb downstream from the peak SNP Bn-A02-p9610453 (*r*^2^ > 0.2, Table S5, Figure 5A). On average, the accessions carrying the major frequency allele of Bn-A02-p9610453 were 8.83 cm taller than those with the alternative allele (Figure 5B). We also identified the GA biosynthesis gene *BnaC07g36630* located at 31.0 kb downstream from the peak SNP Bn-scaff_16069_1-p2212587 (*r*^2^ > 0.4, Table S5, Figure 5C). Overexpression of its ortholog *AtGA2ox8* in *B. napus* caused a 18.8–23.9% reduction in adult plant height (Zhou et al., 2012). In our association panel, accessions carrying the major frequency allele of Bn-scaff_16069_1-p2212587 were 3.48 cm taller than those with the alternative allele (Figure 5D). In addition to the abovementioned candidate genes, we identified other candidates orthologous to documented *Arabidopsis* genes for height regulation, such as *GA1, GA2, GA20ox1, GA20ox2, GA2ox3, GA2ox4, GA2ox6, GAMT1, GID1A, GNL*, and *GNC* (Table S5).

In addition, 25.3% (24/95) of the candidate genes were annotated to associate with BR, and 14.7% (14/95), and 10.5% (10/95) were involved in the BR biosynthesis and signaling pathway, respectively (Table S5). *BnaA04g21780* is orthologous to *AtDET2*, a dwarf gene that participates in the early step of BR biosynthesis (Gao et al., 2008). In our study, *BnaA04g21780* was 11.1 kb downstream from the peak SNP Bn-A04-p16336301 (*r*^2^ > 0.5, Table S5). *BnaC03g68620* is orthologous to another BR anabolic gene, *AtDWF5*, which causes the characteristic BR-deficient dwarf phenotype when mutated (Silvestro et al., 2013). In our study, *BnaC03g68620* was 579.4 kb downstream from the peak SNP Bn-scaff_23761_1-p581335 (*r*^2^ > 0.2, Table S5). We also identified the putative BR signaling gene *BnaA05g12570* located at 701.0 kb upstream from the peak SNP Bn-A05-p9773183 on A05 (*r*^2^ > 0.1, Table S5). A lesion in its ortholog *AtBRI1* causes the characteristic BR-deficient dwarfed stature (Clouse et al., 1996). In addition, we identified other candidates orthologous to the documented *Arabidopsis* genes for height regulation, such as *CYP724A1, CYP90C1, CYP90D1, UGT73C5, STE1, BAK1, BES1, BAS1, BAK1, BIM2*, and *BRL1* (Table S5).

Moreover, we identified candidate genes annotated in other pathways, including auxin (14, 14.7%), polyamine (2, 2.1%), and cytokinin (1, 1.1%; Table S5). *BnaA03g16880* is orthologous to the well-known auxin transporter *AtABCB1*, which produces a dwarf phenotype when mutated (Ye et al., 2013). In the present study, *BnaA03g16880* was located at 41.9 kb upstream from the peak SNP Bn-A03-p8663803 (*r*^2^ > 0.3) and at 166.9 kb downstream from the second strongest signal in XP-CLR analysis (Table S5, Figure 4). Another gene, *BnaA01g07220*, is orthologous to thermospermine oxidase *AtPAO5*, which induces inhibition in stem elongation when its function is lost (Kim et al., 2014). *BnaA01g07220* was found at 30.2 kb upstream from the peak SNP Bn-A01-p3789302 (*r*^2^ > 0.2) and pinpointed in the narrow genome interval of a QTL mapped in TN DH (Table 5). Furthermore, we identified other *Arabidopsis* orthologs for height regulation, such as *ADP1, AGROS, WAT1, BUD2, CLASP, CESA1, IRX8*, and *TCTP* (Table S5).

**Table 5:** **Summary of the GWAS loci and candidate genes pinpointed to narrow genome intervals in the linkage mapping populations**.

**SNP^a^**	**LD^b^**	**Chr**.	**Candidate gene**	**Position (kb)**	**Annotation**	**TN DH (kb)**	**KN DH (kb)**	**GP DH (kb)**	**8M DH (kb)**	**HJ DH (kb)**
Bn-A01-p3789302	170	A01	BnaA01g07220	3406	*PAO5*	3391–3895				
		A01	BnaA01g07300	3433	*ADP1*					
Bn-A02-p9610453	650	A02	BnaA02g12260	6486	*RGA*		2956–9630		6406	6241–7000
Bn-A03-p14611641	250	A03	BnaA03g28010	13,710	*STE1*	13,539-14,039	7914–15,864			
		A03	BnaA03g28050	13,726	*SSI2*					
Bn-A06-p23852270	410	A06	BnaA06g34810	23,009	*RGA*		22,677–23,052			
Bn-A07-p14390667	510	A07	BnaA07g21670	16,750	*WAT1*			16,688–16,913		
Bn-A09-p32172515	370	A09	BnaA09g44210	30,387	*BES1*	30,260–30,766				
Bn-A10-p10830979	210	A10	BnaA10g16110	12,361	*BUD2*		12,612–12,807	12,372–12,611		

a *SNP Only the peak SNP in a defined locus is shown*.

b *LD LD decay rate in units of kb when r^2^ = 0.1*.

## Discussion

The LD decay rate is the primary factor limiting the mapping resolution of GWAS. Previous studies have reported a considerably slower decay rate for the C subgenome than the A subgenome (Qian et al., 2014; Li et al., 2015; Xu et al., 2015; Liu et al., 2016). To further explore the cause of this phenomenon, we performed a genome-wide analysis of the haplotype block structure in our association panel. A recent research in rapeseed has reported the summary information of the haplotype blocks for each chromosome (Qian et al., 2014). However, by adjusting the default settings of Haploview (to compute the LD statistics for all marker pairs), we were able to obtain a more accurate estimation of the haplotype block number, size and physical locations (Table S4). We observed the extensively large pericentromeric haplotype blocks (>3 Mb) throughout the genome, especially in the C subgenome (Table 2). Consistently, the large haplotype blocks on C01 and C04 were also observed in one association panel with 248 winter-type rapeseed accessions (Hatzig et al., 2015). Furthermore, our analysis revealed that these large haplotype blocks were largely blamed for the slower decay rate of the C subgenome than the A subgenome. This result most likely reflects the history of extensively inter-specific crosses between *B. rapa* and *B. napus* in the Chinese breeding program (Qian et al., 2014; Xu et al., 2015; Liu et al., 2016) that has introgressed new allelic diversity into the *B. napus* A subgenome of Chinese accessions. Probably due to the heterochromatic property of pericentromeric regions, which largely suppresses recombination and contributes to the extension of haplotype blocks (Gaut et al., 2007), the large blocks are enriched in pericentromeric regions.

Despite its advantages, association analysis suffers from a strong confounding factor, population structure, which potentially produces spurious associations between traits and loci (Nordborg and Weigel, 2008; Yan et al., 2011). A clear solution to this problem is to complement the association analysis with the linkage analysis, taking advantage of the resolution of the former and the robustness to confounding of the latter (Nordborg and Weigel, 2008). However, the comparison of results between linkage and association analysis is hindered by their different units (centimorgan and base pair, respectively). Moreover, researchers from different labs generally utilize different sets of markers, so it is difficult to compare QTLs from different population maps when the common markers are absent. However, the recently released *B. napus* genome enabled us to determine the physical intervals of the documented QTLs by blasting their flanking markers to the reference genome (Chalhoub et al., 2014). Consequently, we identified the physical intervals of 289 QTLs from nine mapping populations for plant height and found considerable overlaps with our GWAS loci, which provided convincing support to our results (Table S7). Furthermore, eight loci in the present study were pinpointed to the narrow genome intervals (<800 kb, Table 5), which greatly narrowed the search scope for candidate genes.

To reduce the risk of false positives, researchers generally prefer the stringent mixed model, which accounts for kinship and structure (or principal component), to identify the association signals. However, for each of the success stories, there are probably at least as many cases in which mapping efforts were abandoned due to failure in identifying significant signals with the mixed model. For example, no significant SNPs for 12 important traits, such as 100-grain weight and flowering time, were detected by MLM in a GWAS with a panel of 513 densely genotyped maize inbred lines (Yang et al., 2014). In the present study, when using BLUP values across six environments, only three SNPs from two loci were identified above the Bonferroni threshold by the MLM (Figures 3A,B). Similarly, Li et al. identified three loci associated with the plant height BLUP values above the Bonferroni threshold by the MLM (Li et al., 2015). Though Schiessl et al. identified 69 loci associated with plant height with the mixed model (Schiessl et al., 2015), the number decreased dramatically to 10 when using the Bonferroni correction (*P* < 1/n, *n* = 21,623). To identify more loci associated with plant height, on one hand, we used the less stringent FDR correction instead of the stringent Bonferroni correction, which, however, did not lead to any additional discoveries as abovementioned. On the other hand, we included two more permissive models, the GLM and A-D test, in our association analysis. These models have been successfully applied in other studies and have identified multiple plausible loci for target traits with higher power (Yang et al., 2014; Liu et al., 2016). In the present study, these methods succeeded in identifying 66 additional plausible loci for plant height (Table S5, Figures 3C–H). So the number of loci associated with plant height was greatly increased as compared with previous two association studies using the mixed model (Li et al., 2015; Schiessl et al., 2015). One common concern with the utilization of the GLM and A-D test is that they potentially introduce more false positive findings than the MLM. However, our study suggested that the results of these methods are fairly reliable because more than 70% of the significant loci from both methods overlapped intervals of QTLs from previous studies, and plausible candidate genes were identified within the LD decay range up—and downstream of most loci (Table S5). Accordingly, it is feasible to combine the GLM and A-D test with the MLM to maximize the detection power.

To identify the regions of the genome that are most affected by selection during rapeseed semi-dwarf breeding, we used a likelihood method (the cross population composite likelihood ratio, XP-CLR) to scan for extreme allele frequency differentiation over extended linked regions. This method has been successfully utilized to detect the genomic regions conferring favorable phenotypes in other crops, such as the semi-dwarf gene *sd-1* in rice (Xie et al., 2015) and the bitter gene *Bt* in cucumber (Qi et al., 2013). In the present study, we detected multiple GWAS loci as being strongly selected between two subgroups with different plant height (Figure 4). The results thus provided additional evidence to support our GWAS results. However, we failed to detect the selective sweep signatures for nearly two-thirds of the GWAS loci, including considerable loci with large effects and loci harboring established height genes in rapeseed. Even for the loci with selection signals, the XP-CLR scores were fairly low (7.1 on average) when compared with similar analyses in other crops (Qi et al., 2013; Xie et al., 2015). Therefore, it is tempting to speculate that the major height loci of the association panel did not undergo hard selective sweep in historical selection, which coincides with the breeding history of rapeseed; because breeders previously focused on yield, oil seed content and oil quality (double-low canola quality), strong selection was not exerted on the plant-type traits, including plant height. However, to meet the demands of modern agriculture, such as mechanized harvesting, high-density planting, and lodging resistance, the selection for semi-dwarf phenotypes will increase in the succeeding period of time, and these signatures will be reflected in the genome. Because the average distance between two adjacent SNPs (44.4 kb) was much larger than the spacing between two grid points (100 bp) for XP-CLR analysis, some regions of the assembled genome failed to be detected, which presumably led to false negative findings. Therefore, a much higher density of SNP markers is required to reveal more regions under selection for plant height in the future.

Despite the surfeit of mapping publications, few genes have an established role in rapeseed height regulation, which greatly impedes the progress of candidate gene mining in the present study. Encouragingly, the genetic pathways that modify the height of flowering plants are fairly well conserved. For example, mutations in the orthologs of green revolution genes from monocotyledonous plants, including rice *sd-1*, and wheat *Rht*, also caused GA-deficient dwarf phenotypes in the dicotyledonous plant *Arabidopsis* (Peng et al., 1997; Barboza et al., 2013). In addition, ectopic expression of *GAMT1* in the three dicotyledonous plants *Arabidopsis*, tobacco, and petunia all conferred dwarf phenotypes (Varbanova et al., 2007). Furthermore, loss of function of the GA signaling repressor gene *RGA* causes dwarfism in three cruciferous plants, *Arabidopsis, B. rapa*, and *B. napus* (Muangprom et al., 2005; Liu et al., 2010). Hence, referring to publically available literatures on established plant height genes, mainly in the model plant *Arabidopsis*, allowed us to better characterize the candidate genes in rapeseed. These candidate genes are functionally associated with GAs, BRs, auxin, polyamine, and cytokinin (Table S5). GAs, BRs, and auxin are the three major hormones known to have significant effects on plant height regulation (Wang and Li, 2008). In the present study, candidate genes related to the three hormones accounted for more than three-quarters (76.8%) of the total genes (Table S5). In addition to these three hormones, polyamines, a family of low-molecular-weight aliphatic nitrogen compounds, have emerged to further our understanding of plant height control. Loss of function of the S-adenosylmethionine decarboxylase gene *BUD2* and the polyamine oxidase gene *PAO5* leads to reduced plant height in *Arabidopsis* (Ge et al., 2006; Kim et al., 2014). In our research, orthologs of the two genes in rapeseed were located in the narrow confidence intervals of the GWAS loci and QTLs from previous studies (Table 5).

## Author contributions

TF, BY, YZ, CG, JL, and JZ conceived and designed the study. JW, CM, JT, and JS advised on the experimental design. CS, BW, LY, SL, ZZ, and SC performed the phenotyping measurement. CS and KH performed the data analysis. CS wrote the manuscript and all authors reviewed and edited the manuscript.

## Funding

This work is supported by the National High Technology Research and Development Program of China (Grant No. 2012AA101107), the National Basic Research Program of China (2011CB109300), and the 948 Program of the Ministry of Agriculture of China (2011-G23).

### Conflict of interest statement

The authors declare that the research was conducted in the absence of any commercial or financial relationships that could be construed as a potential conflict of interest.
