# Supplementary figures and images for: Genome-Wide Association Study Provides Insight into the Genetic Control of Plant Height in Rapeseed (Brassica napus L.)
Source: Front Plant Sci. 2016 Jul 27;7:1102. doi: 10.3389/fpls.2016.01102 (PMC4961929; doi:10.3389/fpls.2016.01102)

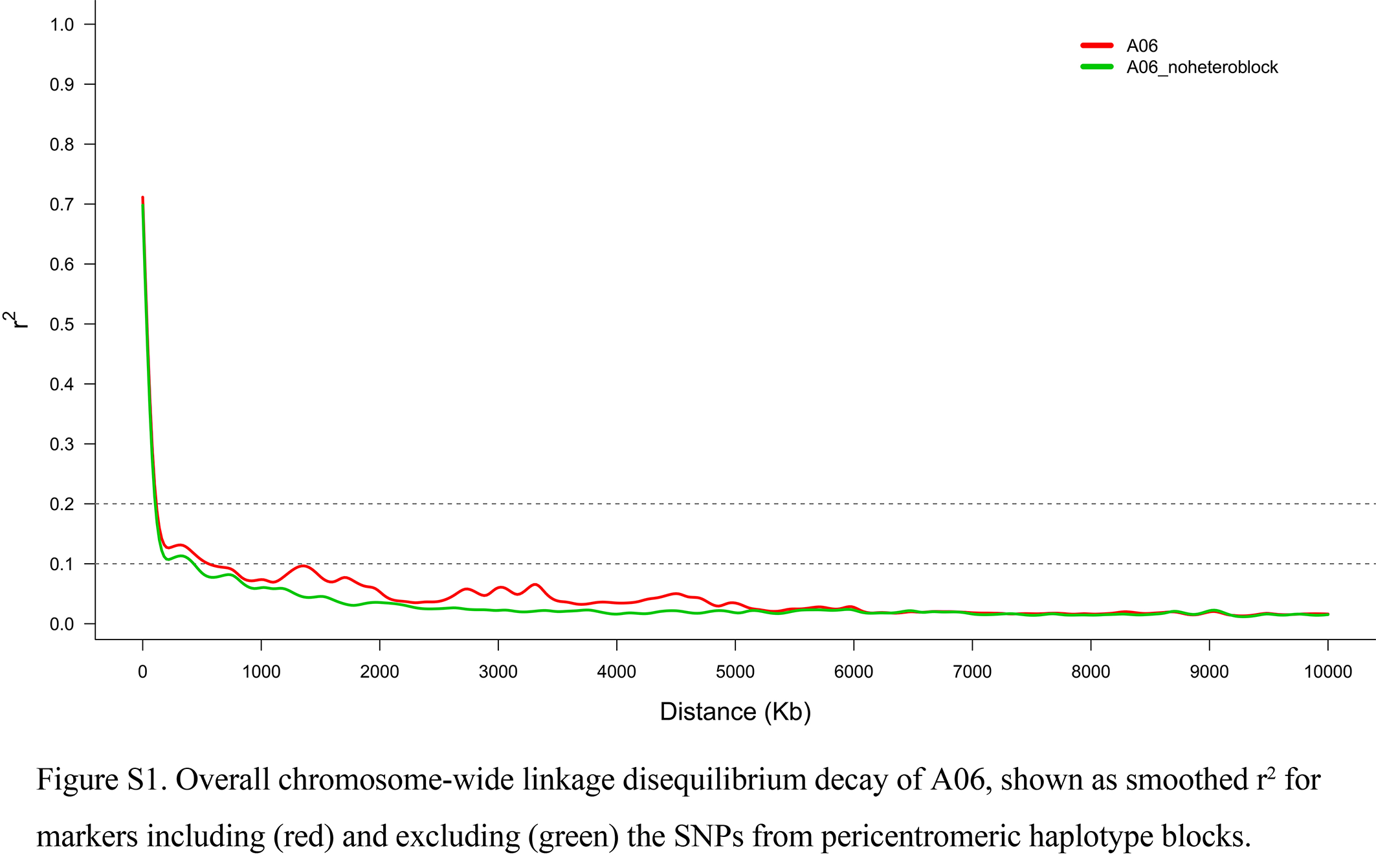

Supplement: Supplementary file 11 [file Image1.TIF]

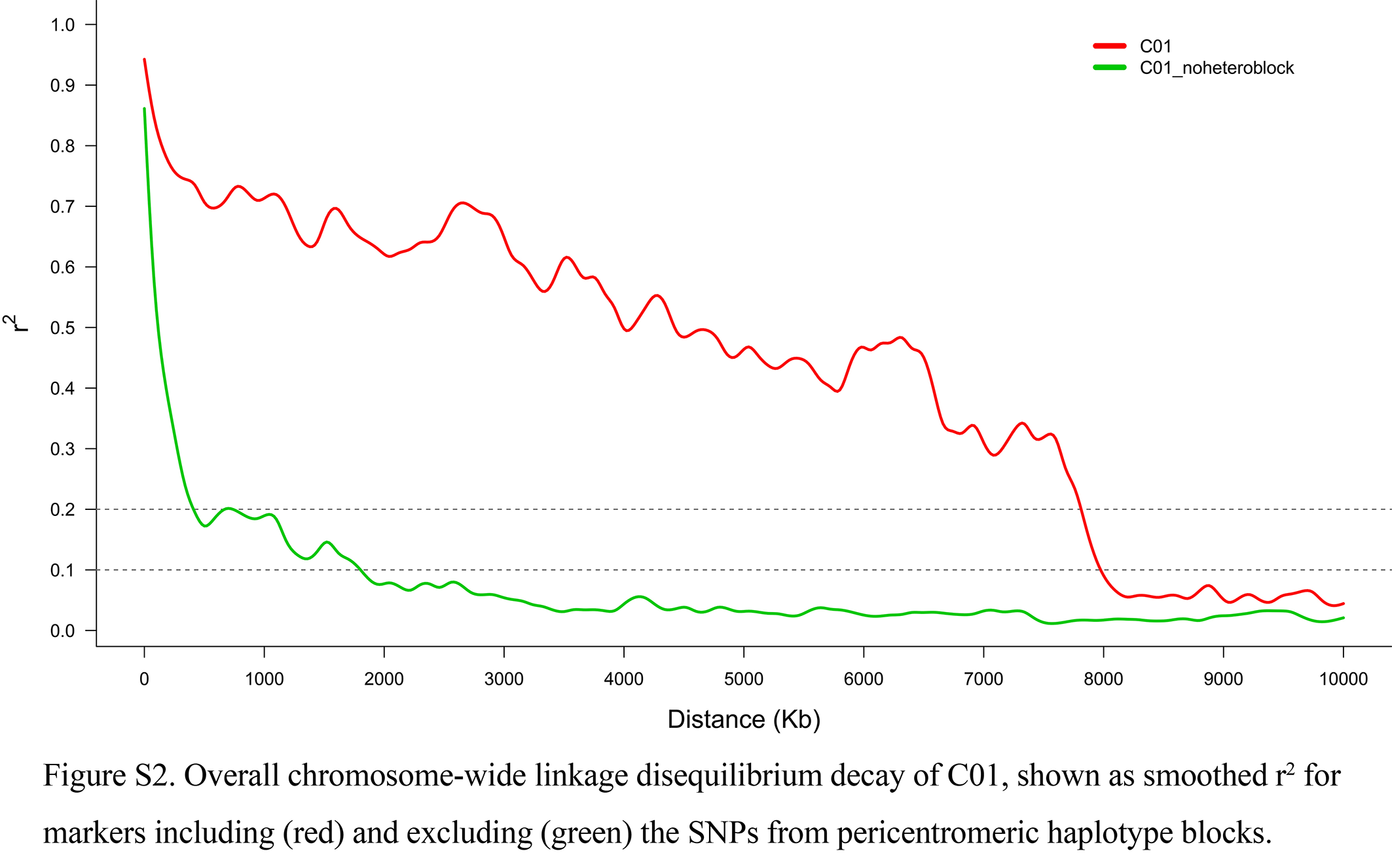

Supplement: Supplementary file 12 [file Image2.TIF]

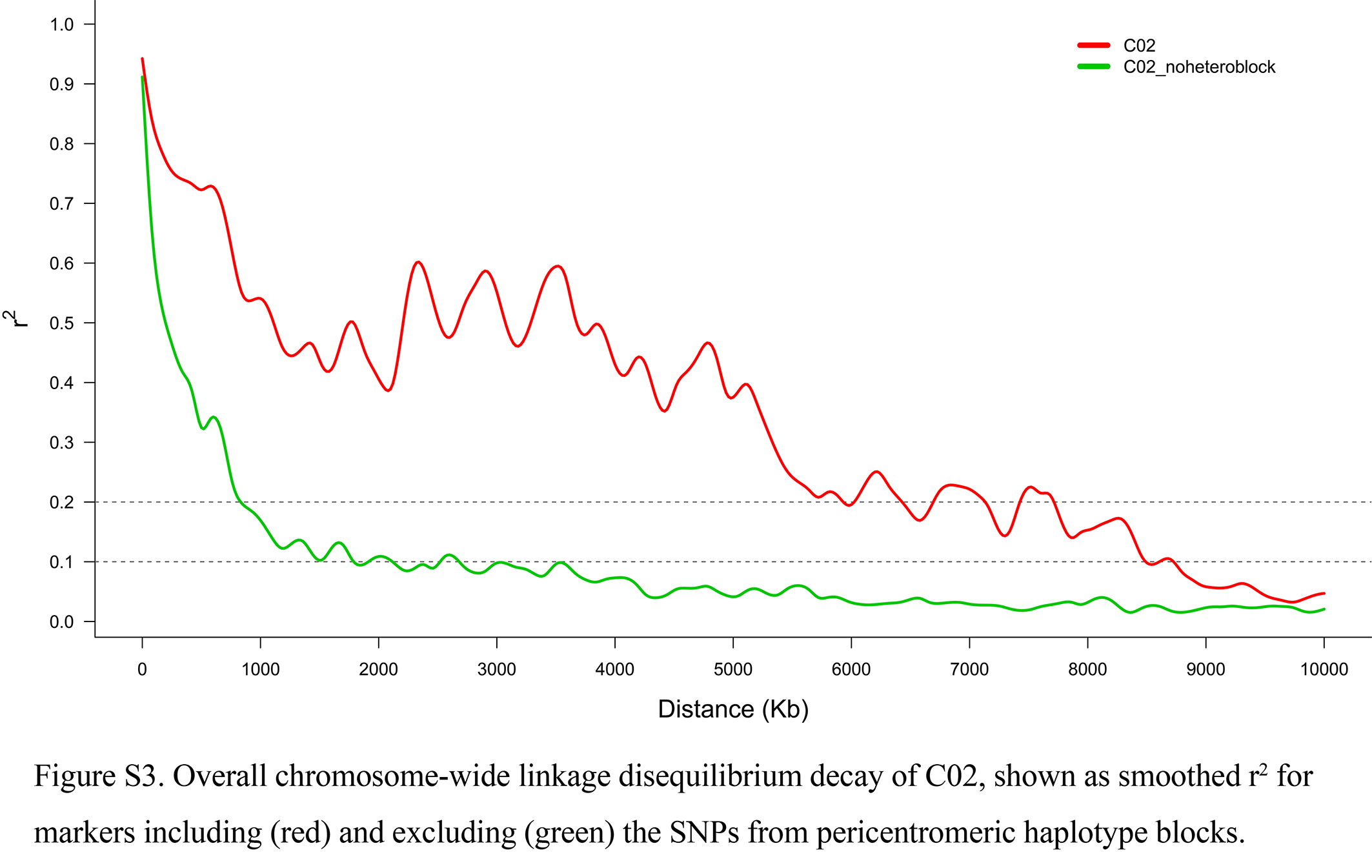

Supplement: Supplementary file 13 [file Image3.TIF]

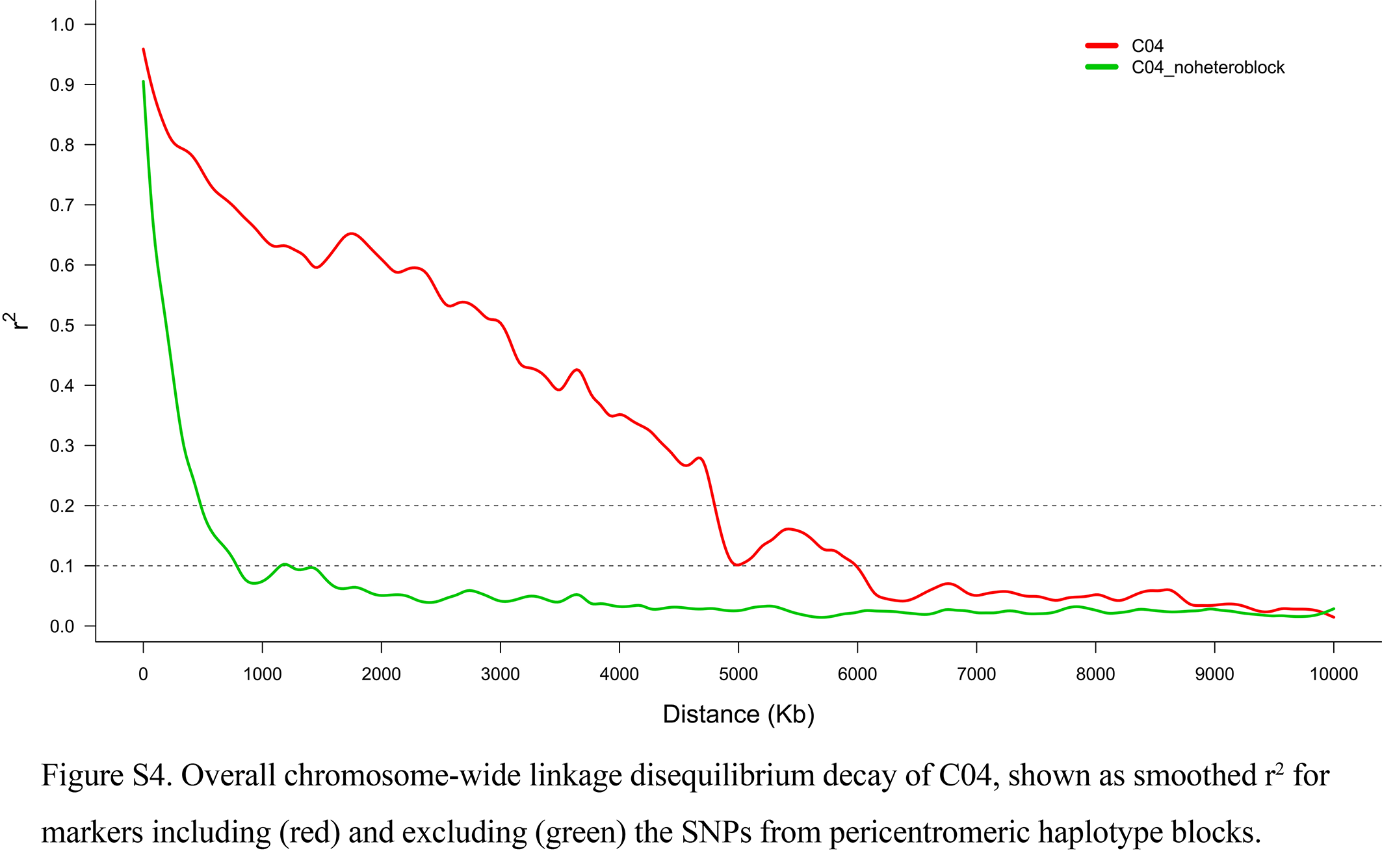

Supplement: Supplementary file 14 [file Image4.TIF]

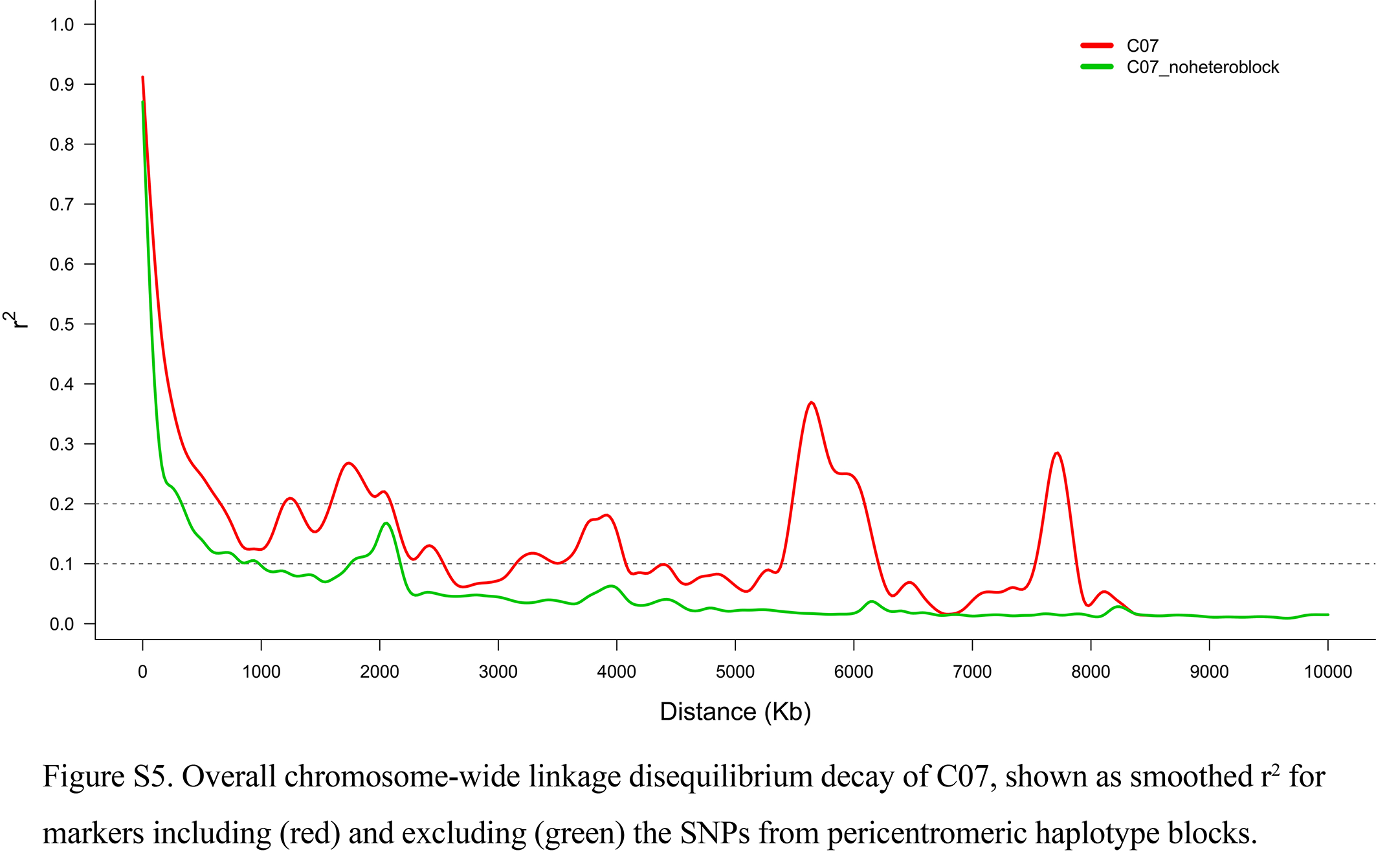

Supplement: Supplementary file 15 [file Image5.TIF]

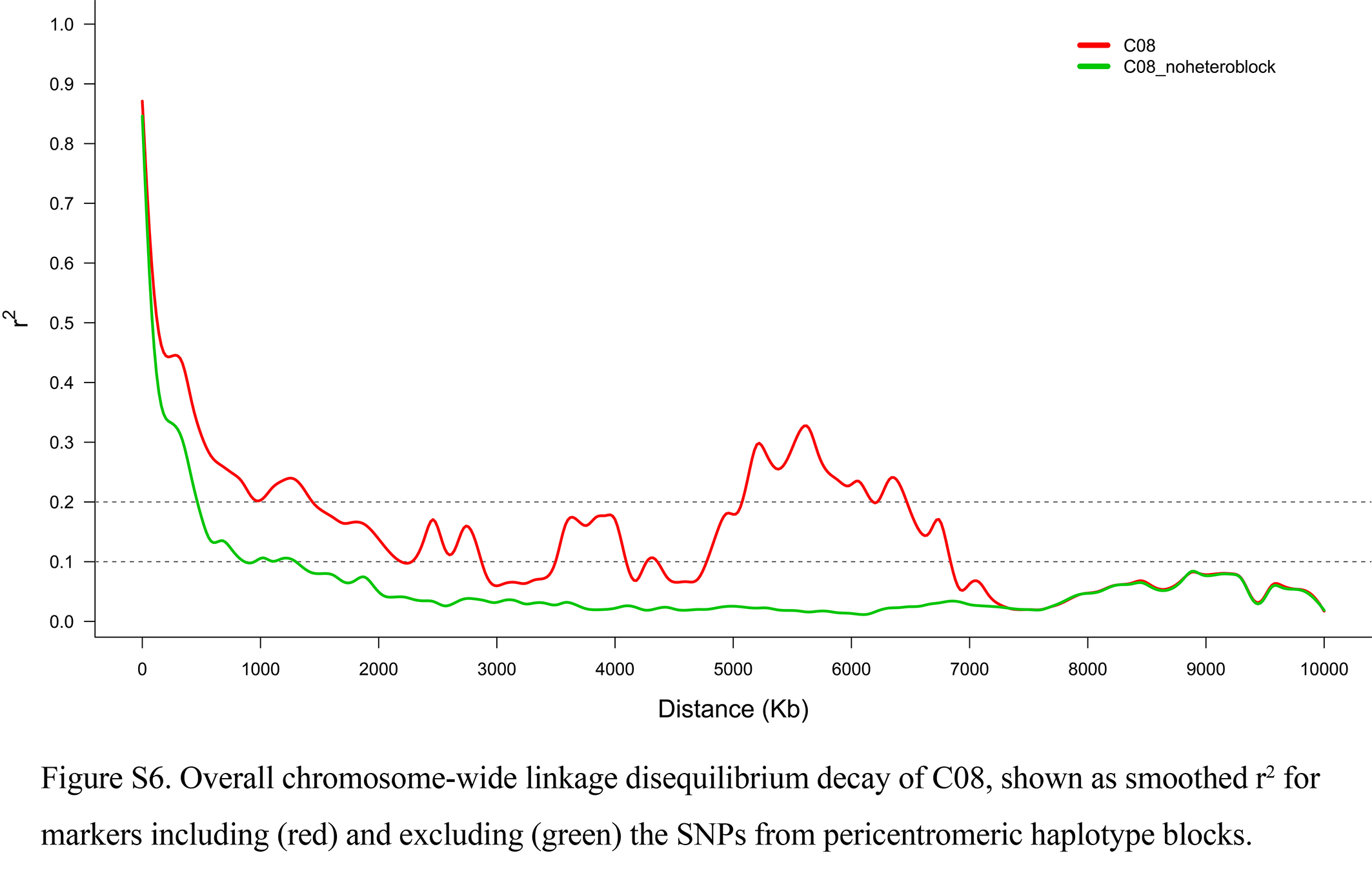

Supplement: Supplementary file 16 [file Image6.TIF]
